# Adalimumab for induction of remission in patients with Crohn's disease: a systematic review and meta-analysis

**DOI:** 10.1186/s40001-022-00817-6

**Published:** 2022-09-30

**Authors:** Juntao Yin, Yang Li, Yangyang Chen, Chaoyang Wang, Xiaoyong Song

**Affiliations:** 1 Department of Pharmacy, Huaihe Hospital, Henan University, Kaifeng, Henan China; 2 Cardiology, Huaihe Hospital, Henan University, Kaifeng, Henan China; 3 General Surgery, Huaihe Hospital, Henan University, Kaifeng, 475000 Henan China; 4 Department of Pharmaceutics, School of Pharmacy, Henan University, Zhengzhou, 450000 Henan China

**Keywords:** Adalimumab, Clinical remission, Crohn’s disease, Systematic review, Meta-analysis

## Abstract

**Purpose:**

A large number of people with Crohn's disease (CD) fail to recover from conventional therapy or biological therapy. Some studies showed that adalimumab (ADA) may be an effective alternative therapy for these patients. The aim of this study was to evaluate the efficacy and safety of ADA in inducing CD remission.

**Methods:**

We performed search of Pubmed/MEDLINE, Embase, CENTRAL, the Cochrane IBD Group Specialized Register, and several other databases. Randomized controlled trials (RCTs) comparing any dose of ADA with controls (placebo or active) in participants with active CD were included. The primary outcome was the failure to achieve clinical response/remission at 4 weeks. Several subgroup and sensitivity analyses were performed. Review Manager Software v5.3 was used.

**Results:**

Four RCTs were included (*n* = 919), in which 553 participants received ADA and 366 participants received placebo. A meta-analysis of four studies showed that at 4 weeks, there were more people in the ADA group with clinical response/remission or symptom improvement compared with the placebo group. The rates of side effects, serious side effects, and study withdrawals due to side effects were lower in ADA participants than placebo ones.

**Conclusion:**

This meta-analysis shows that ADA is superior to placebo in induction of clinical response/remission of CD patients, but no firm conclusions can be drawn on the safety of ADA in CD due to the low number of events.

**Graphical Abstract:**

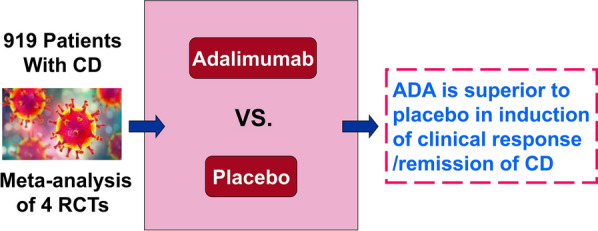

**Supplementary Information:**

The online version contains supplementary material available at 10.1186/s40001-022-00817-6.

## Introduction

Crohn's disease (CD) is a chronic, inflammatory, incurable disease with increasing incidence that can affect any part of the gastrointestinal tract from mouth to the anus [1–4]. CD is associated with significant morbidity and decreased quality of life [5, 6] and the first goal of treatment during this period is clinical response. CD is conventionally treated with systemic corticosteroids or local corticosteroids and immunosuppressives [7]. Gene research may provide important guidance for the clinical treatment of CD [8–10]. However, a significant proportion of patients were subjected to therapy failure with these medications. In addition, more than 50% of patients treated acutely with steroids will either become drug resistant or steroid dependent [11]. These medications are also associated with significant adverse events (AEs). The introduction of novel biologics has changed the landscape of the management of CD [12–14], providing effective and rapid clinical response with minimal AEs. Adalimumab (ADA) has been used to treat moderate-to-severe CD [15–17], and the CLASSIC I trial suggests that ADA may be more effective than placebo in inducing remission in CD patients [18]. Furthermore, a number of trials and meta-analyses have shown no difference between ADA and other biologics, such as infliximab (IFX), certolizumab pegol, golimumab, natalizumab, and vedolizumab, in inducing remission in patients with CD [19–23], but the healthcare costs were significantly lower in ADA users [24] and the rate of AEs was lower [23]. This systematic review will synthesize the available evidence on the efficacy and safety of ADA in inducing CD remission, and will assess the overall quality of the evidence supporting its use.

## Methods

This meta-analysis was conducted in accordance with the criteria established in the Preferred Reporting Items for Systematic Reviews and Meta-Analyses (PRISMA), and a review protocol (CRD42021275088) was registered in the PROSPERO international prospective register of systematic reviews.

### Search strategy

Comprehensive literature searches were performed using Ovid MEDLINE, Embase, The Cochrane Library, The Cochrane IBD Group Specialized Register, Clinicaltrials.gov, and WHO trials registry (ICTRP) (from inception to 5 May 2022). Articles were selected using a highly sensitive search strategy, designed by a librarian (N.W.) and peer reviewed, to identify reports of randomized controlled trials (RCTs), with a combination of MeSH headings and text words that included 1) ADA and synonyms in all fields and 2) inflammatory bowel diseases as a MeSH in titles, abstracts, and journal titles, or Crohn's disease, pancolitis, proctitis, proctocolitis, ulcerative colitis, and gastroenteritis as MeSHs in titles or abstracts. The two concepts were then combined. The searches were run on 5 May 2022 and updated on 30 May 2022. Other available sources of unpublished data (gray literature) were also searched. Recursive searches and cross-referencing were carried out using a “similar articles” function; bibliography of articles identified after an initial search was also manually reviewed.

### Inclusion and exclusion criteria

RCTs assessing the efficacy and safety of ADA for inducing remission in CD were considered to be included. Studies published as abstracts were enrolled only when the authors could be contacted for further information on efficacy and safety outcomes. Participants of any age diagnosed with CD, as defined by conventional clinical, radiological, endoscopic, or histological criteria, were considered for inclusion. Participants must have had active CD at study entry to be included. People with surgically induced remission were excluded. Interventions that involve ADA versus placebo or a control therapy were considered for inclusion.

### Outcomes

The primary outcome was the failure to achieve clinical response/remission at 4 weeks. Secondary outcomes included quality of life, adverse events, serious adverse events, and withdrawals due to adverse events.

### Data collection

Data collection and quality control were done independently by two reviewers (J.Y. and Y.C.). A third reviewer was involved if a conflict occurred. When additional data were required, the first and last authors of the corresponding manuscript were contacted by email with relevant questions; an additional query was sent if no response was received. The extracted data include descriptions of included studies, definition of remission and response, time to remission, time to response, adverse effects, and severe adverse effects. For RCTs, the RoB2 by the Cochrane was used to assess the risk of bias [25].

The methodological quality of enrolled trials was assessed using the Jadad score, which judges descriptions of randomization, blinding, and withdrawals (dropouts) in trials [26]. The quality scale ranges from 0 to 7 points with a low-quality report scoring 3 or less and a high-quality report scoring at least 4 [27].

### Data analysis

In randomized placebo controlled studies, only participants in the treatment arm were analyzed. When several treatment arms using different doses of ADA were reported in the study, we have combined the active treatment groups for analysis.

Data from the included trials were combined for meta-analysis when interventions, participant groups, and outcomes were sufficiently similar (determined by consensus). The pooled *RR* with corresponding 95% CI for dichotomous outcomes was calculated. Heterogeneity among trials was evaluated using the *I*^22^ statistic and the Chi^2^ test [28]. *I*^22^ values of < 25%, 25–50% and  > 50% correspond to low, moderate, and high levels of heterogeneity, respectively. For the Chi^2^ test, a *P* value of 0.10 was considered statistically significant. If no statistical heterogeneity was present, a fixed-effects model was used to pool data. However, if there was heterogeneity (*I*^22^ ranging from 50 to 75%), a random-effects model was used to pool data.

Funnel plot was not used to detect the possibility of publication bias because no sufficient number of studies were included (< 10) in this pooled analysis [29]. Sensitivity analyses were performed based on the quality and weight of the trials, and by excluding each individual trial in turn as recommended by Cochrane Collaboration open learning material for reviewers [30]. Subgroup analyses were also performed based on the interventions in different studies. All statistical analyses were done using RevMan 5.3 (Copenhagen: The Nordic Cochrane Centre, The Cochrane Collaboration, 2008). The PRISMA statement outline for reporting systematic reviews and meta-analyses was used to report this work [31].

## Results

### Study selection

**Fig. 1:**
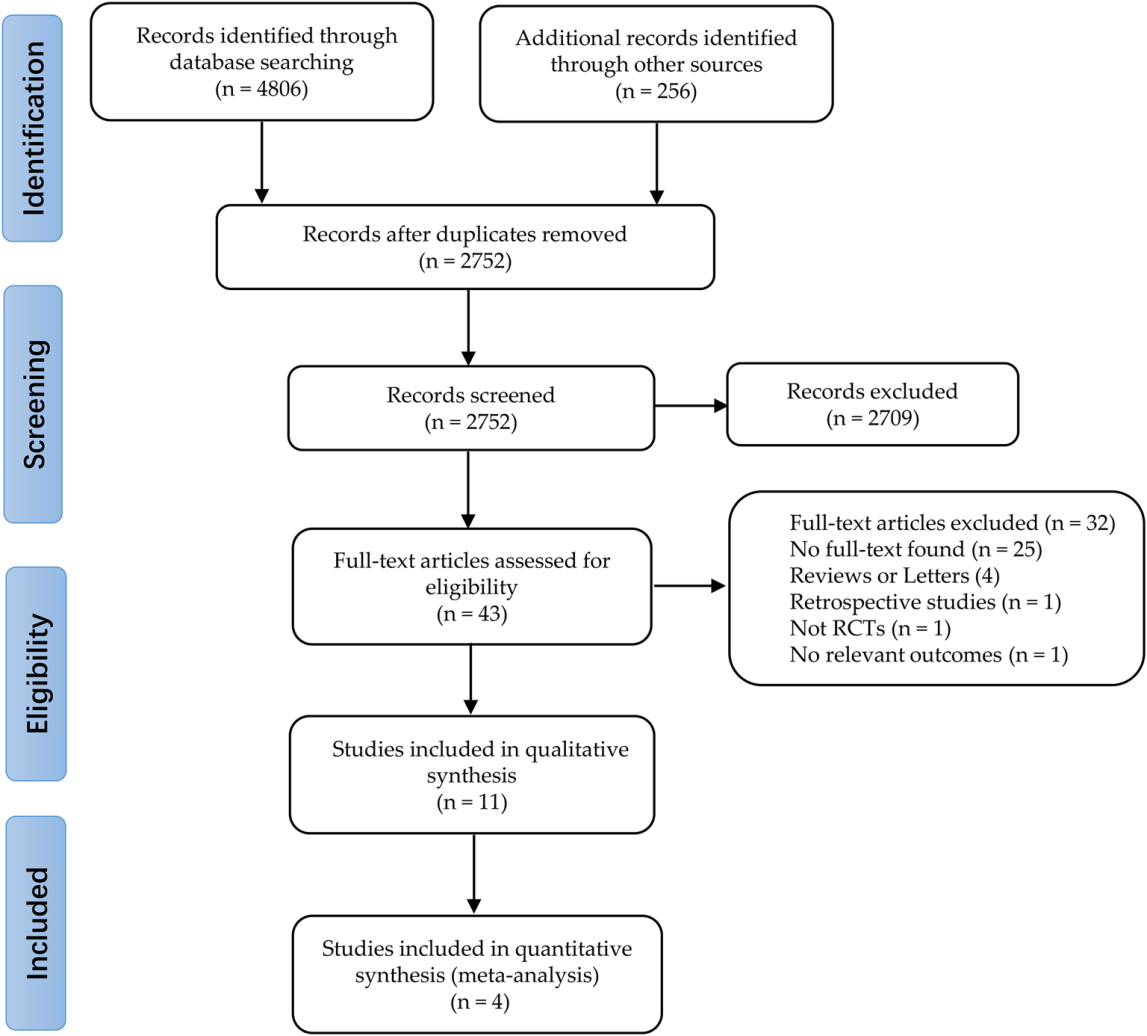
Flow diagram of search strategy and included studies

The retrieval process and results are shown in Fig. 1. A total of 4 out of 1820 identified studies (*n* = 919 patients, including 553 in the ADA group and 366 in the placebo group), were included [18, 32–34], all were performed between 2006 and 2020. And all studies were published in English. There was excellent inter-reviewer agreement [Kappa = 0.82 (95% CI: 0.72–0.92)]. The characteristics of included studies are shown in Table 1. The first author of one study provided further data after being contacted by the authors [34].

**Table 1:** Characteristics of included studies

Descriptions of included studies						Outcomes available for analysis				
N	Study	Funding	Design	Main criteria	inclusion N	Definition of remission	Definition of response		Induction of Remission wk	Induction of Response wk
1	Chen 2020	Yes	RCT	CDAI > 220 Anti-TNF naive	102	CDA < 150	ΔCDAI > 70	ΔCDAI > 100	4	4
2	Hanauer 2006	Yes	RCT	CDAI > 220 Anti-TNF naive	225	CDA < 150	ΔCDAI > 70	ΔCDAI > 100	4	4
3	Sandborn 2007	Yes	RCT	CDAI 220–450, IFX resistant	159	CDA < 150	ΔCDAI > 70	ΔCDAI > 100	4	4
4	Watanabe 2012	Yes	RCT	CDAI > 220	67	CDA < 150	ΔCDAI > 70	ΔCDAI > 100	4	4

### Failure to achieve clinical remission at 4 weeks

**Fig. 2:**
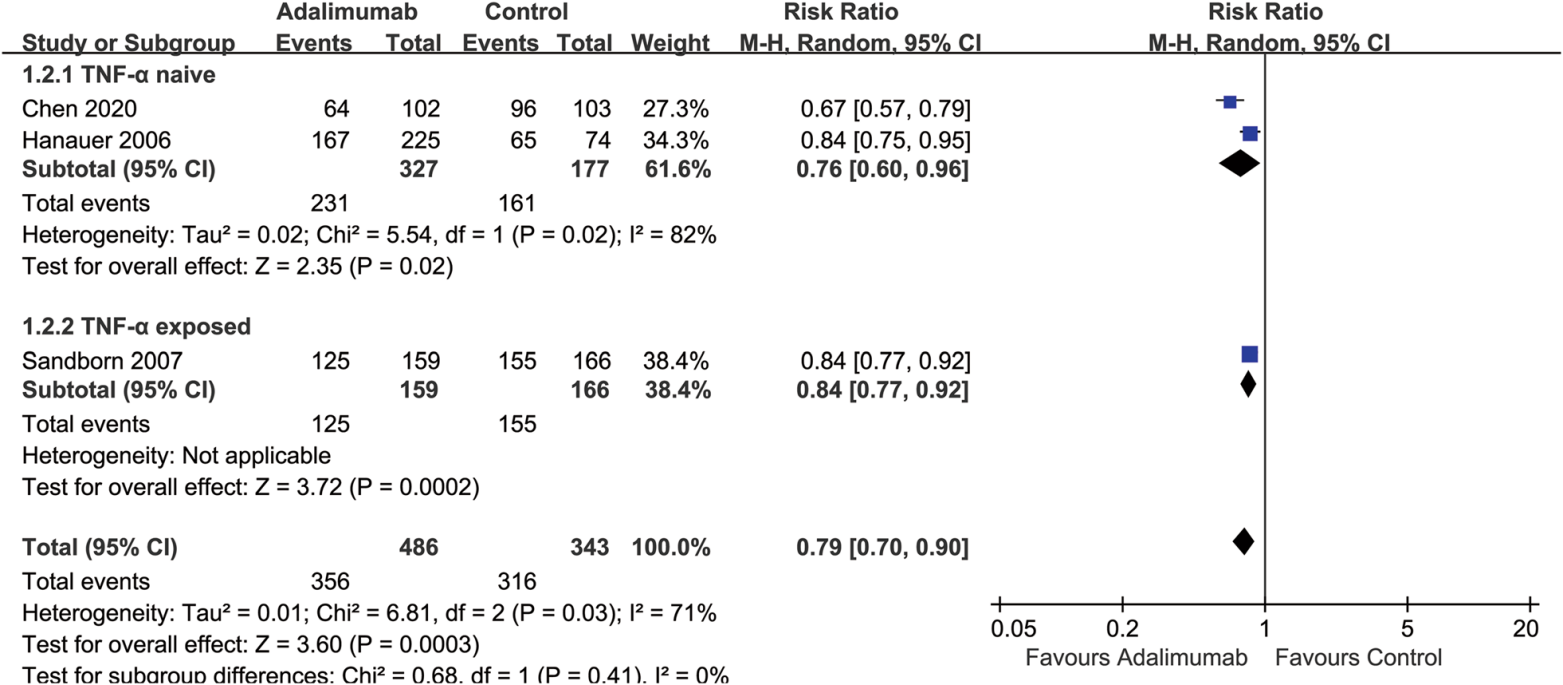
Forest plot for failure to achieve clinical remission at 4 weeks in ADA and control groups

All of the included studies (919 participants) reported on clinical remission (CDAI < 150) at 4 weeks [18, 32–34]. The results showed that the ADA group had a higher clinical remission rate compared with placebo group (Fig. 2). Seventy-three percent (406/553) of ADA participants failed to achieve clinical remission at 4 weeks compared with 92% (336/366) of placebo participants (*RR* 0.81, 95% CI 0.73 to 0.90, *I*^22^ = 53%) (Fig. 2). A sensitivity analysis based on a random-effects model produced similar results (*RR* 0.81, 95% CI 0.73 to 0.90) (Fig. 2). Similarly, a sensitivity analysis excluding the study assessed to be at unclear risk of bias produced similar results (*RR* 0.79, 95% CI 0.70 to 0.90) (Additional file 1: Figure S1).

In the group of 160 mg/80 mg dose, 70% (260/370) of participants in the ADA group failed to achieve clinical remission compared with 93% (283/305) of placebo participants (*RR* 0.76, 95% CI 0.66 to 0.86, *I*^22^ = 56%) (Fig. 2). A subgroup analysis based on concomitant aminosalicylates was performed. In the group of 160 mg/80 mg dose, 70% (260/370) of participants in the concomitant aminosalicylates group failed to achieve clinical remission compared with 93% (283/305) of non-concomitant aminosalicylates participants (*RR* 0.81, 95% CI 0.67 to 0.98, *I*^22^ = 76%) (Additional file 1: Figure S2). In the 80 mg/40 mg dose group, 78% (85/109) of participants in the ADA group failed to achieve clinical remission compared with 89% (32/36) of placebo participants (*RR* 0.87, 95% CI 0.74 to 1.01, *I*^22^ = 2%) (Fig. 2). Lastly, in the 40 mg/20 mg dose group, 82% (61/74) of participants in the ADA group compared with 84% (21/25) of placebo participants (*RR* 0.98, 95% CI 0.80 to 1.20) (Fig. 2). Overall, the 160 mg/80 mg dose group appeared to be the most effective for inducing clinical remission at 4 weeks, and concomitant aminosalicylates group was more effective than non-concomitant aminosalicylates group. However, the test for subgroup differences by dose showed no difference between the dose subgroups (test for subgroup differences Chi^2^ = 5.00, *P* = 0.08, *I*^22^ = 60.0%) (Fig. 2).

Two studies [18, 32] enrolled participants who were TNF-α naïve and one study [33] enrolled participants who had been treated previously with infliximab, which allowed for a subgroup analysis by previous exposure to TNF-α. Among TNF-α-naive participants, 71% (231/327) of the ADA group failed to achieve clinical remission at 4 weeks compared with 91% (161/177) of the placebo group (*RR* 0.76, 95% CI 0.60 to 0.96) (Additional file 1: Figure S1). Among TNF-α exposed participants, 79% (125/159) of the ADA group failed to achieve clinical remission at 4 weeks compared with 93% (155/166) of the placebo group (*RR* 0.84, 95% CI 0.77 to 0.92) (Additional file 1: Figure S1). Test for subgroup differences showed no difference between TNF-α exposure subgroups (test for subgroup differences Chi^2^ = 0.08, *P* = 0.41, *I*^22^ = 0%) (Additional file 1: Figure S1).

### ***Failure to achieve clinical response at ***4 ***weeks***

#### 70-point clinical response

**Fig. 3:**
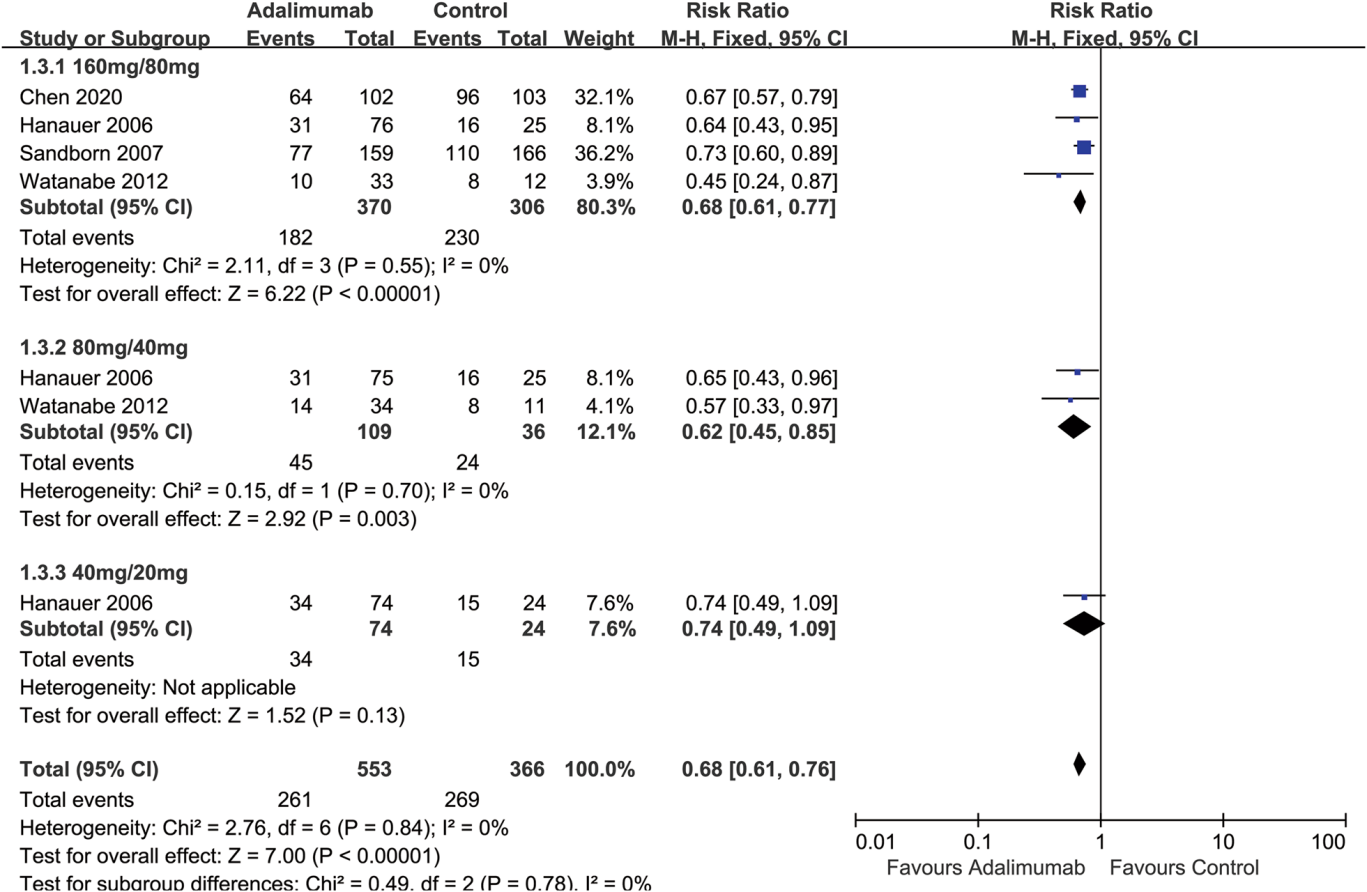
Forest plot for failure to achieve clinical response at 4 weeks (70-point response) subgroup by dose in ADA and control groups

All of the included studies [18, 32–34] (919 participants) reported on clinical response defining a reduction of 70 points in the CDAI score. Forty-seven percent (261/553) of ADA participants failed to achieve a 70-point clinical response at 4 weeks compared with 73% (269/366) of placebo participants (*RR* 0.68, 95% CI 0.61 to 0.76, *I*^22^ = 0%) (Fig. 3).

The results showed that the 160 mg/80 mg and 80 mg/40 mg dose groups had a higher 70-point clinical response rate compared with the placebo group (Fig. 3). In the 160 mg/80 mg dose group, 49% (182/370) in the ADA group failed to achieve a 70-point clinical response compared with 75% (230/306) of the placebo group (*RR* 0.68, 95% CI 0.61 to 0.77, *I*^22^ = 0%). In the group of 80 mg/40 mg dose, 41% (45/109) in the ADA group failed to achieve a 70-point clinical response compared with 67% (24/36) of the placebo group (*RR* 0.62, 95% CI 0.45 to 0.85, *I*^22^ = 0%). Lastly, in the group of 40 mg/20 mg dose, 46% (34/74) of ADA participants failed to achieve a 70-point clinical response compared with 63% (15/24) of the placebo group (*RR* 0.74, 95% CI 0.49 to 1.09). Overall, the 160 mg/80 mg dose group appeared to be the most effective for the induction of a 70-point clinical response after 4 weeks of treatment. However, the test for subgroup differences by dose showed no difference between the dose subgroups (test for subgroup differences Chi^2^ = 0.49, *P* = 0.78, *I*^22^ = 0%) (Fig. 3).

Among TNF-α-naive participants, 49% (160/327) of the ADA group failed to achieve a 70-point clinical response at 4 weeks compared with 81% (143/177) of the placebo group (*RR* 0.67, 95% CI 0.59 to 0.77) (Additional file 1: Figure S3). Among TNF-α exposed participants, 48% (77/159) of the ADA group failed to achieve a 70-point clinical response at 4 weeks compared with 66% (110/166) of the placebo group (*RR* 0.73, 95% CI 0.60 to 0.89) (Additional file 1: Figure S3). The test for subgroup differences showed no difference between TNF-α-naive and TNF-α exposure subgroups (test for subgroup differences Chi^2^ = 0.48, *P* = 0.49, *I*^22^ = 0%) (Additional file 1: Figure S3).

#### 100-point clinical response

**Fig. 4:**
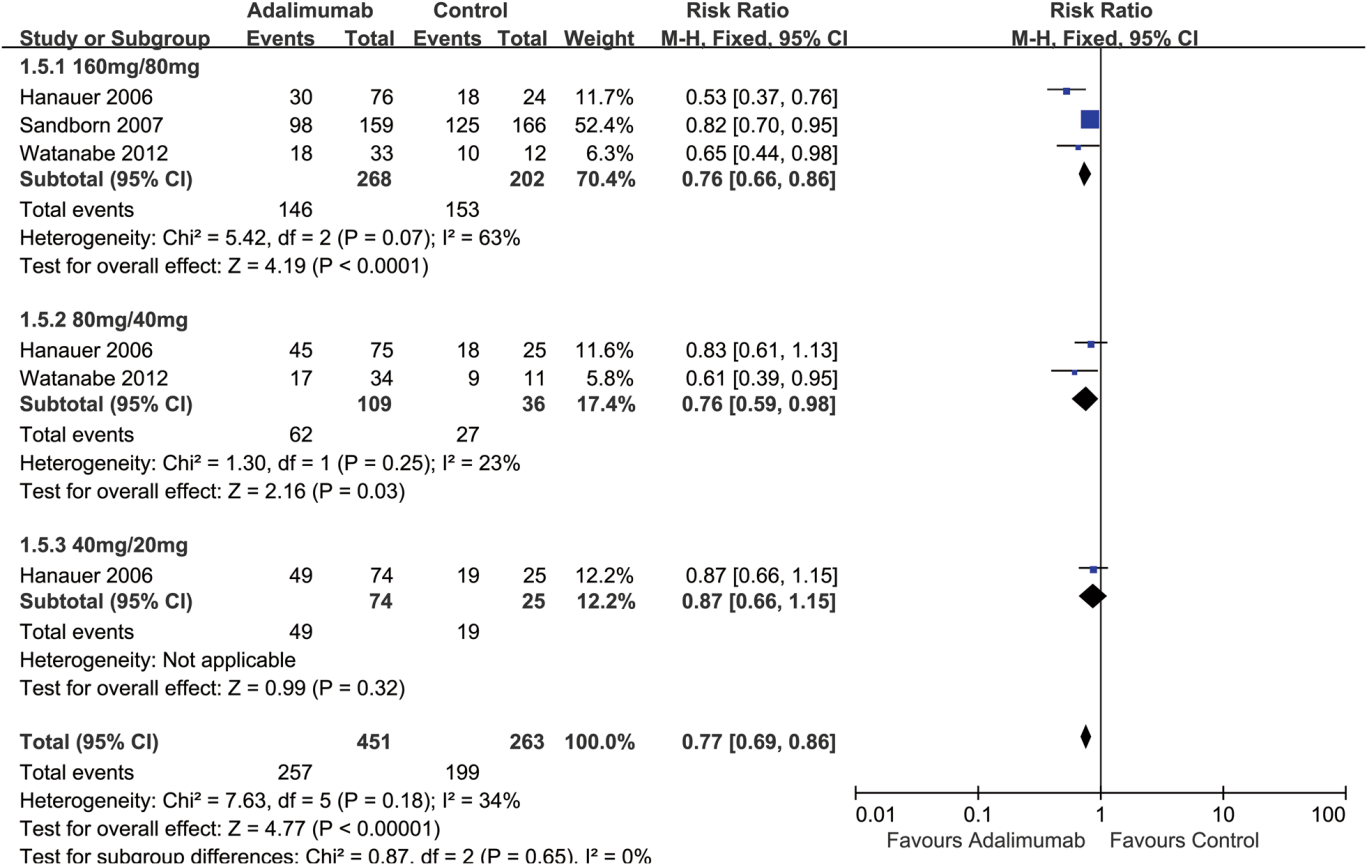
Forest plot for failure to achieve clinical response at 4 weeks (100-point response) subgroup by dose in ADA and control groups

Only three of the included four studies (714 participants) assessed clinical response defined as a 100-point reduction in the CDAI score [18, 33, 34], and the other one [32] (205 participants) did not assess this outcome. The results showed that the ADA group had a higher 100-point clinical response rate compared with the placebo group. Fifty-seven percent (257/451) of ADA participants failed to achieve a 100-point clinical response at 4 weeks compared with 76% (199/263) of participants in the placebo group (*RR* 0.77, 95% CI 0.69 to 0.86, *I*^22^ = 0%) (Fig. 4).

The rates of 100-point clinical response were different across the three dose groups. In the 160 mg/80 mg dose group, 54% (146/268) of the ADA group failed to achieve a 100-point clinical response compared with 76% (153/202) of the placebo group (*RR* 0.76, 95% CI 0.66 to 0.86, *I*^22^ = 0%) (Fig. 4). In the 80 mg/40 mg dose group, 57% (62/109) of the ADA group failed to achieve a 100-point clinical response compared with 75% (27/36) of the placebo group (*RR* 0.76, 95% CI 0.59 to 0.98, *I*^22^ = 23%) (Fig. 4).

Lastly, in the 40 mg/20 mg dose group, 66% (49/74) of the ADA group failed to achieve a 100-point clinical response compared with 76% (19/25) of the placebo group (*RR* 0.87, 95% CI 0.66 to 1.15) (Fig. 4). Overall, the 160 mg/80 mg dose group appeared to be the most effective for inducing a 100-point clinical response following 4 weeks of treatment. However, the test for subgroup differences by dose showed no difference between the dose subgroups (test for subgroup differences Chi^2^ = 0.87, *P* = 0.65, *I*^22^ = 0%) (Fig. 4).

Among TNF-α naïve participants, 55% (124/225) of the ADA group failed to achieve a 100-point clinical response at 4 weeks compared with 74% (55/74) of the placebo group (*RR* 0.74, 95% CI 0.62 to 0.89) (Additional file 1: Figure S4). Among TNF-α exposed participants, 62% (98/159) of the ADA group failed to achieve a 100-point clinical response at 4 weeks compared with 75% (125/166) of the placebo group (*RR* 0.82, 95% CI 0.70 to 0.88) (Additional file 1: Figure S4). The test for subgroup differences showed no difference between TNF-α-naive and TNF-α exposure subgroups (test for subgroup differences Chi^2^ = 0.69, *P* = 0.41, *I*^22^ = 0%) (Additional file 1: Figure S4).

### Quality of life

All of the included studies [18, 32–34] (919 participants) reported on quality of life as an outcome. Chen et al. [32] reported significantly higher IBDQ scores at week 4 in the ADA 160 mg/80 mg group compared with the placebo group (*P* < 0.01). Hanauer et al. [18] reported significantly higher IBDQ scores at week four in the ADA 160 mg/80 mg and 80 mg/40 mg dose groups compared with the placebo group. Sandborn et al. [33] reported a mean IBDQ score at week 4 of 150 in the ADA group compared with 139 in the placebo group (*P* < 0.001). Watanabe et al. [34] reported that SF-36 scores were significantly higher in ADA 160 mg/80 mg and 80 mg/40 mg dose groups at week four compared with the placebo group. Watanabe et al. [34] also reported higher IBDQ scores at week four in the adalimumab160 mg/80 mg dose group compared with the placebo group, although the differences were not statistically significant. For this outcome a narrative synthesis was conducted, and the estimates were not precise.

### Adverse events

**Fig. 5:**
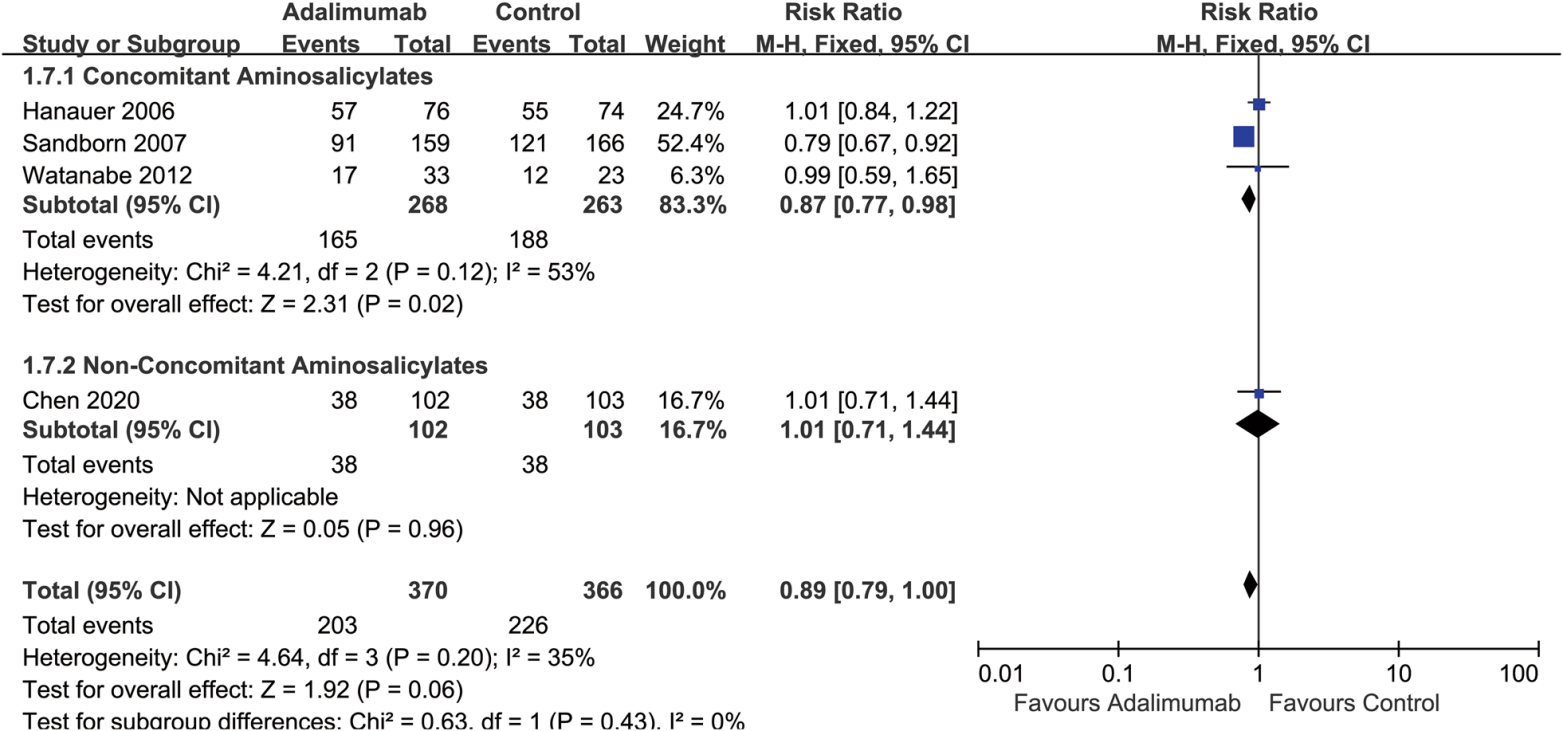
Forest plot for adverse events in ADA and control groups

All of the included studies (919 participants) reported on the proportion of participants that developed at least one AE. Fifty-five per cent (203/370) of the ADA group experienced at least one AE compared with 62% (226/366) of the placebo group (*RR* 0.89, 95% CI 0.79 to 1.00, *I*^22^ = 35%) (Fig. 5). Additionally, in the 160 mg/80 mg dose group, 55% (203/370) of participants in the concomitant aminosalicylates group experienced at least one AE compared with 62% (226/366) of non-concomitant aminosalicylates group (*RR* 0.89, 95% CI 0.79 to 1.00, *I*^22^ = 35%) (Additional file 1: Figure S5). The most commonly reported AEs included injection site reactions, gastrointestinal (GI) symptoms (nausea, and abdominal pain), fatigue, and deterioration of CD [35, 36].

### Serious adverse events

**Fig. 6:**
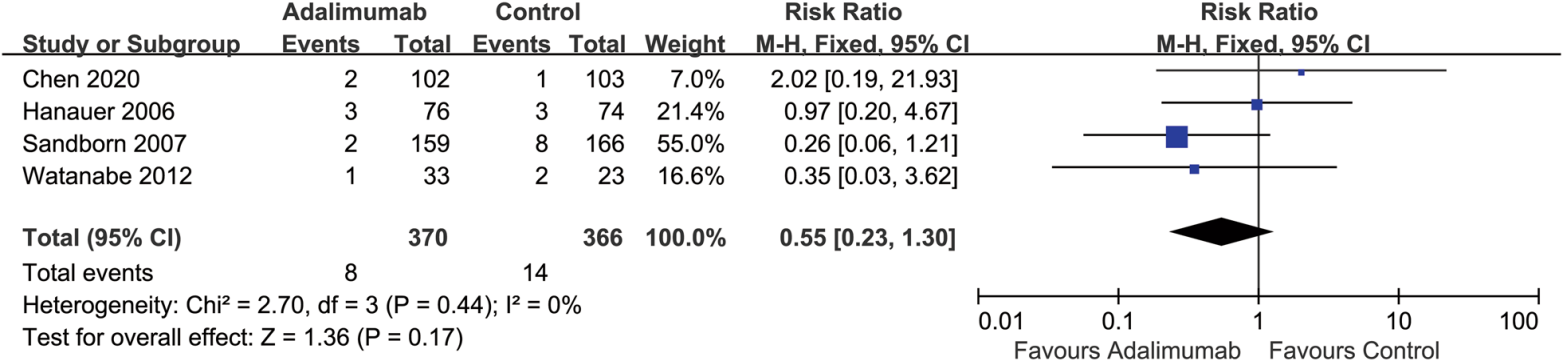
Forest plot for serious adverse events in ADA and control groups

All of the included studies (919 participants) reported on the proportion of participants that developed at least one serious adverse event (SAE). Two percent (8/370) of ADA participants experienced at least one SAE compared with 4% (14/366) participants in the placebo group (*RR* 0.55, 95% CI 0.23 to 1.30, *I*^22^ = 0%) (Fig. 6). The most commonly reported SAEs were related to the underlying CD including infections, CD flares, abscesses, and dehydration.

### Withdrawals due to adverse events

All of the included studies (919 participants) reported on withdrawals due to AEs. Two per cent (6/370) of ADA participants withdrew due to an AE compared with three per cent (12/366) of participants in the placebo group (*RR* 0.51, 95% CI 0.20 to 1.29; *I*^22^ = 0%) (Additional file 1: Figure S6). AEs that led to study withdrawal included worsening CD, abdominal or liver abscesses.

### Other outcomes and analyses

The included studies did not report endoscopic remission, endoscopic response, histologic remission, histologic response, and steroid withdrawal. The data did not allow for planned subgroup analyses based on disease duration, disease severity, disease extent, concomitant medication, and route of administration. None of the included studies had a loss of greater than 10% in follow-up rates; thus, no prespecified sensitivity analysis based on best-case versus worst-case scenarios was undertaken. None of the included studies was published as an abstract only; thus, no prespecified sensitivity analysis based on full-text report versus abstracts or unpublished studies was undertaken.

### Risk of bias and quality of evidence assessment

The risk of bias, according to the RoB 2: A revised Cochrane risk-of-bias tool for randomized trials [37], identified little concern. Of the 4 studies, three were of high quality, and only one study was rated as at unclear risk of bias. Details of the risk assessment are shown in Additional file 1: Figure S7. No funnel plot was used to assess for potential publication bias, because only four studies were included in the pooled analysis [29].

## Discussion

In this meta-analysis, we have demonstrated that treating participants with ADA is better than treating them with placebo for inducing remission and improving symptoms in patients with moderately to severely active CD. Our findings demonstrate that ADA is superior to placebo for induction of clinical remission at 4 weeks, and ADA was more effective than placebo for inducing 70-point and 100-point clinical response at 4 weeks. The 160 mg/80 mg dose group is commonly used and subgroup analysis suggested that the dose may be most effective in inducing clinical remission and clinical response [38]. However, it revealed no significant differences by dose, perhaps owing to the small sample size. The optimal dose of ADA needs to be determined by further research. Although concomitant medications for patients with moderate-to-severe CD are often effective [39], these medications are commonly associated with risks [40, 41]. To determine the efficacy and safety of concomitant medications, we performed a subgroup analysis and concluded that concomitant aminosalicylates group was superior to non-concomitant aminosalicylates for induction of clinical remission at 4 weeks.

CLASSIC I trial [18] included biologic-naive participants, and at week 4 the remission rate in the ADA group was 36% compared with 12% in the placebo group. Similar remission rates were observed in the other three studies. Although population were randomized to groups, it is observed that the effect sizes of 160 mg/80 mg dose were inconsistent in different studies. The reduction of hs-CRP and FC shown in ADA group indicates objective biologic evidence of reduced inflammation. These results are consistent with those shown in the ADA study EXTEND, which showed effects on mucosal healing, although the endoscopy was not performed [42]. Even though CD patients previously exposed to TNF-α were more likely to have a refractory phenotype [22, 43] our subgroup analysis suggested that previous exposure to TNF-α may have no effect on the efficacy of ADA, as efficacy rates were similar between the TNF-α-naive and TNF-α exposed subgroups. The overall incidence of AEs in ADA group during double-blind period was similar to that in the placebo group, and the overall safety profile observed was similar to those observed among other studies [44]. Strikingly, comprehensive data from global CD studies manifested that serious infection rate was 6.7/100 patient-years [44].

Four other systematic reviews that assessed the efficacy and safety of ADA in CD patients were identified [45–48]. Abbass et al. [45] assessed the efficacy and safety of ADA in patients with moderately to severely active CD, which included three RCTs and concluded that ADA was effective for achieving short-term clinical response or remission in CD participants [18, 33, 34]. Huang et al. [46, 47] also assessed the efficacy and safety of ADA in inducing and maintaining remission of participants with moderate-to-severe CD, which included four RCTs and concluded that ADA was effective and significantly improved the life quality of CD participants [18, 33, 49, 50]. Song et al. [47] assessed the efficacy and safety of ADA in patients with moderate-to-severe CD, which included six RCTs and the results showed that ADA was effective for achieving short-term clinical response or remission, long-term remission, and complete fistula healing in participants with CD [18, 33, 34, 42, 49, 51]. Singh et al. [48] concluded that ADA might be preferred as a second-line therapy (after infliximab loss of response), for induction of clinical remission in patients with moderate-to-severe CD. All of these reviews reached the same conclusion as revealed by us. However, they included maintenance trials, long-term clinical response or remission, besides induction trials. The present study covers the largest cases (involving four RCTs with 919 patients). Furthermore, we performed a subgroup analysis of concomitant medications and concluded that concomitant aminosalicylates group was similar to non-concomitant aminosalicylates.

Three of the four included studies were judged as low risk of bias [18, 32, 33], and the other one [34] was assessed to be unclear risk of bias due to random sequence generation, allocation concealment, and blinding. The overall certainty of evidence for the primary outcome (clinical remission at 4 weeks) was rated as moderate due to high heterogeneity (*I*^22^ = 53%), and the overall certainty of evidence for the secondary outcome (clinical remission of previous TNF-α exposure participants at 4 weeks) was also rated as moderate due to high heterogeneity (*I*^22^ = 71%). This heterogeneity may be due to the different subgroups of ADA dose. The overall certainty of evidence for AEs was rated as high, and the overall certainty of evidence for SAEs and withdrawal due to AEs was low possibly due to inconsistency of judgment criteria.

Comprehensive literature search was conducted in order to reduce selection bias. In addition, two authors independently screened, extracted data, and assessed the study quality to minimize bias. Even though we had included four RCTs, we could not adequately control important confounding factors, such as disease phenotype and smoking. The limitation of this systematic review lies in the small sample size and the sparse data. Although the long-term efficacy and safety of ADA treatment in CD patients has been confirmed [34, 52–54], there are still some adverse reactions have been reported [23, 55–57]. The short follow-up time is also a limitation because the included studies only assessed outcomes at 4 weeks, which may not have been enough to capture AEs related to ADA. Further research is required to assess the effect of ADA on these outcomes.

## Conclusion

Our meta-analysis suggests that ADA is superior to placebo for induction of clinical remission and response in patients with moderately to severely active CD. AEs, SAEs, and withdrawals due to AEs were lower in ADA participants compared with placebo. However, we are uncertain about the effect of ADA on AEs due to the low number of events. Therefore, no firm conclusions can be drawn regarding the safety of ADA in CD. Further studies with more participants are required to assess the long-term efficacy and safety of ADA in CD participants, and future RCTs should more clearly assess AEs.

## Supplementary Information

**Additional file 1: Figure S1. **Forest plot for failure to achieve clinical remission at 4 weeks subgroup by previous TNF-α exposure in ADA and control groups.

**Additional file 2: Figure S2.** Forest plot for failure to achieve clinical remission at 4 weeks subgroup by concomitant medication in ADA and control groups.

**Additional file 3: Figure S3.** Forest plot for failure to achieve clinical response (70-point response) at 4 weeks subgroup by previous TNF-α exposure in ADA and control groups.

**Additional file 4: Figure S4.** Forest plot for failure to achieve clinical response (100-point response) at 4 weeks subgroup by previous TNF-α exposure in ADA and control groups.

**Additional file 5: Figure S5.** Forest plot for adverse events at 4 weeks by concomitant medications.

**Additional file 6: Figure S6.** Forest plot for withdrawals due to adverse events in ADA and control groups

**Additional file 7: Figure S7.** Results of the Cochrane Risk of Bias assessment tool 2 for randomized controlled trials

**Additional file 8: Table S1.**

## Data Availability

The dataset used during the study are available from the corresponding author on a reasonable request.

## Abbreviations

RCT: Randomized controlled studies
CDAI: Crohn's disease activity index
IFX: Infliximab
