# Supplementary material for: Adalimumab for induction of remission in patients with Crohn's disease: a systematic review and meta-analysis
Source: Eur J Med Res. 2022 Sep 30;27:190. doi: 10.1186/s40001-022-00817-6 (PMC9523983; doi:10.1186/s40001-022-00817-6)
Supplement: Supplementary file 8 — Additional file 8: Table S1. [file 40001_2022_817_MOESM8_ESM.doc]

**List of Excluded Studies (n = 32)**

**No full-text found (n = 25)**

1. Bossa F, Annese V, Scimeca D, Biscaglia G, Colombo E, Martino G, et al. Infliximab versus adalimumab in Crohn's disease patients in daily clinical practice. a prospective single center experience. Digestive and Liver Disease 2012;44:S115-6.

2. Crandall W, Grifiths A, Colletti R, Ruemmele F, Faubion WW, Hyams JS, et al. Steroid-free remission in adalimumab-treated pediatric patients with moderately to severely active Crohn's disease in the Imagine 1 trial. Journal of Gastroenterology and Hepatology 2014;29:150.

3. Dubinsky M, Hyams JS, Rosh J, Markowitz J, Ruemmele F, Eichner S, et al. Impact of disease duration on clinical outcomes with adalimumab treatment in patients from Imagine 1. United European Gastroenterology Journal 2014;1:A81.

4. Dubinsky M, Rosh J, Faubion WA, Kierkus J, Ruemmele F, Hyams J, et al. Rate of and response to dose escalation in paediatric patients with Crohn's disease from Imagine 1. Journal of Crohn's and Colitis 2014;8:S218.

5. Dubinsky M, Rosh JR, Faubion WA, Kierkus J, Ruemmele F, Hyams JS, et al. Rate of and response to dose escalation in pediatric patients with Crohn's disease from Imagine 1. Gastroenterology 2014;1:S26.

6. Wu KC, Ran ZH, Gao X, Chen M, Zhong J, Sheng JQ, et al. Adalimumab therapy achieves clinical remission and response at week 26 in Chinese patients with Crohn's disease. Inflammatory Bowel Diseases 2016;22:S43.

7. Grifiths A, Baldassano R, Bronsky J, Hyams J, Lazar A, Hyams J, et al. Improvement in markers of bone metabolism with adalimumab in children with moderately to severely active Crohn's disease: Results from Imagine 1. American Journal of Gastroenterology 2013;108:S622.

8. Grifiths A, Baldassano R, Walters T, Bronsky J, Hyams J, Lazar A, et al. Improvement in markers of bone metabolism with adalimumab in children with moderately to severely active Crohn's disease: results from Imagine 1. United European Gastroenterology Journal 2013;1:A528.

9. Grifiths A, Crandall W, Colletti R, Ruemmele F, Faubion, WA, Hyams J, et al. Steroid-free remission in adalimumab-treated paediatric patients with moderately to severely active Crohn's disease in the Imagine 1 trial. Journal of Crohn's and Colitis 2014;8:S12.

10. Grifiths AM, Crandall W, Colletti RB, Ruemmele F, Faubion WA, Hyams JS, et al. Steroid-free remission in adalimumab-treated pediatric patients with moderately to severely active Crohn's disease in the Imagine 1 trial. Gastroenterology 2014;1:S213.

11. Hyams J, Rosh J, Markowitz J, Kierkus J, Dubinsky M, Turner D, et al. Effect of adalimumab on clinical laboratory parameters in paediatric Crohn's disease patients from Imagine 1. Journal of Crohn's and Colitis 2015;9:S275.

12. Hyams J, Ruemmele F, Colletti R, Kierkus J, Rosh J, Eichner S, et al. Impact of concomitant immunosuppressant use on adalimumab efficacy in children with moderately to severely active Crohn's disease: results from Imagine 1. Journal of Crohn's and Colitis 2014;8:S257-8.

13. Hyams JS, Dubinsky M, Ruemmele F, Rosh J, Eichner S, Maa JF, et al. Baseline factors associated with therapeutic response to adalimumab in pediatric patients with Crohn's disease: Data from Imagine 1. Journal of Pediatric Gastroenterology and Nutrition 2016;63:S229-31.

14. Hyams JS, Rosh JR, Markowitz J, Kierkus J, Dubinsky M, Turner D, et al. Effect of adalimumab on clinical laboratory parameters in pediatric Crohn's disease patients from Imagine 1. Gastroenterology 2015;1:S638-9.

15. Hyams JS, Ruemmele F, Colletti RB, Kierkus J, Rosh J, Eichner S, et al. Impact of concomitant immunosuppressant use on adalimumab efficacy in children with moderately to severely active Crohn's disease: results from Imagine 1. Journal of Crohn's and Colitis 2014;8:S430.

16. Hyams JS, Ruemmele F, Colletti RB, Kierkus J, Rosh JR, Eichner S, et al. Impact of concomitant immunosuppressant use on adalimumab efficacy in children with moderately to severely active Crohn's disease: results from Imagine 1. Gastroenterology 2014;1:S214.

17. Hyams JS, Grifiths A, Markowitz J, Baldassano RN, Faubion Jr WA, Colletti RB, et al. Safety and efficacy of adalimumab for moderate to severe Crohn's disease in children. Gastroenterology 2012;143(2):365-74.

18. Kierkus J, Crandall W, Hyams JS, Rosh J, Markowitz J, Baldassano R, et al. Early response to treatment with adalimumab in children with moderately to severely active Crohn's disease: results from Imagine 1. United European Gastroenterology Journal 2013;1:A530.

19. Kierkus J, Crandall W, Hyams J, Rosh J, Markowitz J, Baldassano R, et al. Early response to treatment with adalimumab in children with moderately to severely active Crohn's disease: results from Imagine 1. American Journal of Gastroenterology 2013;103:S621-2.

20. Ruemmele F, Dubinsky M, Hyams JS, Eichner S, Maa JF, Lazar A, et al. Efficacy and safety of adalimumab in pediatric patients with Crohn's disease aged 10 years and younger: subanalysis of Imagine 1. Gastroenterology 2016;1:S588.

21. Ruemmele F, Rosh J, Colletti R, Faubion W, Markowitz J, Eichner S, et al. Efficacy of adalimumab for treatment of perianal fistula in children with moderately to severely active Crohn's disease: results from Imagine 1. United European Gastroenterology Journal 2013;1:A16-7.

22. Turner D, Hyams J, Dubinksy M, Faubion W, Eichner S, Li Y, et al. Relationship of the pediatric Crohn's disease activity index (PCDAI) and Crohn's disease activity index (CDAI) in Imagine 1. United European Gastroenterology Journal 2015;1:A439.

23. Turner D, Hyams JS, Dubinsky M, Faubion WA, Eichner S, Li Y, et al. Relationship of the pediatric Crohn's disease activity index (PCDAI) and Crohn's disease activity index (CDAI) in Imagine 1. Gastroenterology 2015;1:S640.

24. Walters T, Faubion WA, Grifiths A, Baldassano R, Escher J, Ruemmele F, et al. Growth improvement in adalimumab-treated paediatric patients with Crohn's disease: data from Imagine 1. United European Gastroenterology Journal 2014;1:A230.

25. Walters TD, Faubion WA, Grifiths AM, Baldassano RN, Escher J, Ruemmele FM, et al. Adalimumab treatment improves linear growth in children with moderately to severely active Crohn's disease: results from the Imagine 1 trial. Journal of Pediatric Gastroenterology and Nutrition 2016;63:S369-70.

**Reviews or Letters (n = 4)**

26. Nuti F, Fiorino G, Danese S. Adalimumab for the treatment of pediatric Crohn's disease. Expert Rev Clin Immunol. 2015;11:963-72.

27. Abbass M, Cepek J, Parker CE, Nguyen TM, MacDonald JK, Feagan BG, et al. Adalimumab for induction of remission in Crohn's disease. Cochrane Database Syst Rev. 2019;2019:CD012878.

28. Cassinotti A, Ardizzone S, Porro GB. Adalimumab for the treatment of Crohn's disease. Biologics. 2008;2:763-77.

29. Zimmerman L, Bousvaros A. The pharmacotherapeutic management of pediatric Crohn's disease. Expert Opin Pharmacother. 2019;20:2161-68.

**Retrospective studies (n = 1)**

30. Rosh JR, Lerer T, Markowitz J, Goli SR, Mamula P, Noe JD, et al. Retrospective Evaluation of the Safety and Effect of Adalimumab Therapy (RESEAT) in pediatric Crohn's disease. Am J Gastroenterol. 2009;104:3042-9.

**Not RCTs (n = 1)**

31. Bouhnik Y, Carbonnel F, Laharie D, Stefanescu C, Hébuterne X, Abitbol V, et al. Efficacy of adalimumab in patients with Crohn's disease and symptomatic small bowel stricture: a multicentre, prospective, observational cohort (CREOLE) study. Gut. 2018;67:53-60.

**No relevant outcomes (n = 1)**

32.Hyams JS, Crandall W, Rosh JR, Ruemmele F, Escher JC, Lazar A, et al. Efficacy and safety of standard vs low dose adalimumab maintenance therapy as a function of disease severity in pediatric patients with Crohn's disease: subanalysis of Imagine 1. Gastroenterology 2013;1:S887.
